# Association between Active Travel to School and Depressive Symptoms among Early Adolescents

**DOI:** 10.3390/children7050041

**Published:** 2020-05-02

**Authors:** Jiayi Gu, Si-Tong Chen

**Affiliations:** 1 School of Physical Education and Sport, Shanghai Normal University, Shanghai 200234, China; 2 Department of Physical Education, Shenzhen University, Shenzhen 518061, China; sitongchen@szu.edu.cn

**Keywords:** active school travel, adolescents, mental health, depressive symptoms, China

## Abstract

Background: Although much evidence has demonstrated the positive relationship of active school travel (AST) and physical health, little is known about the relationship of AST and mental health indicators among early adolescents, especially in Chinese populations. Thus, this study aimed to investigate the relationship of AST with depressive symptoms and its sex as well as age difference among early adolescents from Shanghai urban areas, China. Methods: 6478 adolescents (mean age = 13.6) in urban area were recruited, of whom boys accounted for 46.2%. A self-reported questionnaire in Chinese was used to collect data on AST and depressive symptoms, and other control variables. Multivariable logistic regression analyses were used to explore the relationships of AST with depressive symptoms. Results: Of all included participants, 53.2% of adolescents reported being active in AST without sex difference. The prevalence of depressive symptoms was 19.2% without sex difference. AST was associated with reporting no depressive symptoms in adolescents (adjusted OR = 1.20, 95%CI: 1.06–1.36). However, the relationship was significant in boys (adjusted OR = 1.34, 95%CI: 1.11–1.60), in those who were grade 8 (adjusted OR = 1.25, 95%CI: 1.01–1.55) and 9 (adjusted OR = 1.29, 95%CI: 1.01–1.65) adolescents. Conclusions: AST may play an important role in preventing depressive symptoms among early adolescents. However, the relationship of AST with depressive symptoms differed by sex and age. More research is encouraged to explore the mechanism linking AST and depressive symptoms among adolescents, especially in different contexts.

## 1. Introduction

Depressive symptoms are part of a type of emotional disorder, with typical symptoms including feelings of sadness, sleep loss, and suicidal thoughts that have a significantly adverse impact on people’s health [1,2]. Globally, more than 264 million people of all ages suffer from depressive symptoms, so this has become an alarming mental health issue [2]. In China, data show that approximately 20% of Chinese children and adolescents exhibit depressive symptoms [3], and the proportions vary by region or city, in the range of 10%–50% [4]. Indeed, depressive symptoms are a serious health issue that has aroused the attention of policymakers in China. The Healthy China Blueprint 2019–2030 has engendered evidence-based information in an effort to alleviate the health burdens of depressive symptoms and prevent them in children and adolescents [5]. A reduction in depressive symptoms requires the need for joint efforts from multiple sectors. Within this framework, epidemiologists from physical activity (PA) behavior must play a vital role in preventing and decreasing depressive symptoms in children and adolescents with the preventive effects from PA behaviors.

Well-established evidence demonstrated that increased PA can reduce the risk factors associated with depressive symptoms in young people [6,7,8]. For example, engaging in more PA was associated with lower depressive symptoms in Norwegian adolescents [9]. This finding was replicated among Chinese adolescents [6]. In addition, links between increased PA and the decrease of depressive symptoms was supported by evidence from systematic reviews [7,10]. Evidential research suggests that promoting PA may be a feasibly efficient approach to prevent depressive symptoms in adolescents, even though its underlying mechanism remains fully unanswered (neurobiologically or psychologically) [7,10]. On this point, as an important source of PA [11], active school travel (AST) may be a way against depressive symptoms. There is evidence concerning the relationship of AST with depressive symptoms among adults [12]. As a result, it is assumed and expected that AST may play a role in preventing depressive symptoms among adolescents. If researchers confirm a significant relationship between AST and depressive symptoms, economic mental health preventions or interventions can be accomplished, which, in turn, more feasibly and efficiently lightens adolescents’ mental health burden.

A growing number of studies have recently focused on the role of AST on mental wellbeing indicators (e.g., depressive symptoms) among young people [13]. Theoretically, AST can improve overall PA, which can reduce depressive symptoms, offering a foundation to prevent depressive symptoms among adolescents. As a daily behavior because of schooling, adolescents can more easily accumulate PA with AST [13]. In this regard, AST can be used to prevent depressive symptoms among adolescents. In an integrative review by Waygood et al. [13], AST was shown to lead to positive emotions and reduce negative emotions in young people because of many reasons, like social interactions or physical environmental feedbacks. Biddle et al. [7] found that, owing to the many varied contexts of PA, more exploration is needed on the different types of PA associated with improving mental health among young people. The concept of sustainable happiness has indicated that school travel mode is related to emotion and wellbeing [14] because positive psychology is interdependent on lifestyle from diverse aspects. Therefore, sustainable behavior, for instance, walking and cycling, has the potential to promote better mental health in young people.

However, across the literature, many studies have focused on the role of AST in promoting physical and behavioral health [11], while lacking in evidence on the contributions of AST to mental wellbeing among adolescents. It is understood that only two previous studies have examined the relationship of AST with mental health indicators. For example, a study using a nationally representative sample suggested that children and adolescents with AST were less likely to be depressed in China [15]. Another study by Canadian researchers indicated that AST was positively related with positive emotions in elementary school students [16]. Those findings support that AST may contribute to better mental health indicators among children and adolescents. However, such evidence is currently insufficient [13]. Such inadequate evidence on the relationship between AST and mental health indicators cannot fully support policies or strategies to prevent depressive symptoms among adolescents. Another consideration is regional or cultural differences. Waygood et al. [13] suggested that regional and cultural differences are possible barriers in replicating the association between AST and mental health indicators; thus, consistent findings were identified as either culturally or regionally specific by more studies. Clearly, more research needs to be done to confirm any positive influence of AST on mental health. 

In addition, an important gap in the literature still exists. A systematic review has demonstrated that the effects of PA on mental health may be influenced by sex and age among adolescents [7]. For example, a study among Chinese adolescents found that sufficient PA can be a protective role on depressive symptoms in boys of 14 years old or older; however, such a significant relationship was also observed in adolescent girls [17]. A recent European adolescent study indicated that the relationship of being active with being less depressive in boys was stronger than that in girls (Odds ratio (OR) for boys = –1.7 vs. OR for girls = –0.7) [18]. Indeed, there are gender and age differences in AST behavior among adolescents [11] that may lead to differences in the relationship of AST with depressive symptoms. Collectively, it is assumed that the relationship of AST with depressive symptoms differs by sex and age. However, little is known about the relationship of AST with mental health and sex and age [15,16]. This uncertainty is an obstacle to designing more effective prevention for mental health issues. Consequently, whether sex and age differences are significantly contributing factors for AST with depressive symptoms among adolescents needs to be explored. 

Thus, to address these evidence gaps across the literature, the current study aims to explore the relationship of AST with depressive symptoms among early adolescents with a sample from Shanghai, China taking sex and age differences into account.

## 2. Methods

### 2.1. Study Survey 

The survey was conducted at public middle schools in Shanghai (urban area), China between March and June 2018. Thirty middle schools from eight districts within Shanghai were invited to participate in the survey, and a total of 26 schools agreed to join the survey. In each selected school, the survey was conducted in three randomly selected classes from Grades 7 to 9. In total, 8190 participants were invited to participate in the survey. A total of 7469 students completed the survey; a response rate of 91.2%. Ultimately, 6478 participants (age: mean = 13.7, standard deviation = 0.9) provided valid data on all pertained variables for this study. During the survey, student privacy was protected through anonymous and voluntary participation. In addition, informed consent was obtained from the students, parents, and/or school officials. The study and data collection were approved by the Human Research Ethics Committee of Shanghai Normal University (approval code 2018030016). Participants in this study and their parents or legal guardians provided written consent to engage in the survey. The Global School-based Student Health Survey questionnaire (Chinese version) was adopted to conduct data collection. All participants were required to fill in this survey questionnaire in their classrooms during school recess on schooldays.

### 2.2. Measures

#### 2.2.1. AST (Exposure)

All participants were required to answer the question “How many days did you walk or ride a bicycle to and from school during the past 7 days?” Participants self-reported their responses according to their actual situations (responses: 0–7 days). Participants reporting 7 days of walking or cycling were regarded as active AST participants, whereas those not reporting 7 days of walking or cycling were regarded as passive AST participants, in line with previous studies [19]. This measure was similar to that used in the study by Fulton et al. [19] that was validated with acceptable reliability [20].

#### 2.2.2. Depressive Symptoms (Outcomes)

Depressive symptoms were assessed with the question “During the past 12 months, did you ever feel so sad or hopeless almost every day for two weeks or more in a row that you stopped doing your usual activities?” Responses were listed as (1) yes and (2) no. Participants answering affirmatively to this question were considered to have depressive symptoms [21,22].

#### 2.2.3. Control Variables

On the basis of previous studies, we included the variables of grade (age) [17,21], sex [17,23,24], screen time [17,22,25], smoking [9], drinking [9], friends [26,27], and weight status (BMI: body mass index) [28,29]. Participants’ information was collected using a self-reported questionnaire containing the following questions: (1) grade (“What is your current grade?” Responses: 1 = 7th, 2 = 8th, 3 = 9th); (2) sex (“What is your sex?” Responses: 1 = male, 2 = female); (3) screen time (“How much time do you spend during a typical or usual day sitting and watching television, playing computer games, talking with friends, or doing other sitting activities such as playing the piano, playing cards, or reading books?” Responses: 1 = less than 1 hour per day, 2 = 1–2 hours per day, 3 = 3–4 hours per day, 4 = 5–6 hours per day, 5 = 7–8 hours per day, 6 = more than 8 hours per day; participants reporting 3 to 4 hours per day and above were regarded as excessive); (4) smoking status (“During the past 30 days, on how many days did you smoke cigarettes?” Responses: 1 = 0 days, 2 = 1 or 2 days, 3 = 3–5 days, 4 = 6–9 days, 5 = 10–19 days, 6 = 20–29 days, 7 = all 30 days); (5) alcohol consumption (“During the past 30 days, on how many days did you have at least one drink containing alcohol?” Responses: 1 = 0 days, 2 = 1 or 2 days, 3 = 3–5 days, 4 = 6–9 days, 5 = 10–19 days, 6 = 20–29 days, 7 = all 30 days); (6) number of close friends (responses: 1 = 0, 2 = 1, 3 = 2, 4 = 3 or more); (7) height (cm) and weight (kg) were self-reported, from which we calculated the body mass index (BMI). According to the BMI, the Chinese age- and sex-specific overweight/obesity cut-off [30] was used to determine participants’ weight status, which has been used widely in studies of young Chinese populations [31,32,33].

### 2.3. Statistical Analysis

In statistical analysis, AST and depressive symptoms were transformed into binary variables (AST: passive vs. active; depressive symptoms: no vs. yes). Some control variables were converted into the binary variable, including sedentary time (ST, limited: ≤2 hours per day vs. excessive: >2 hours per day) according to current recommendations [33]; the variable of weight status was treated as non-obesity vs. obesity. The variables of smoking and drinking were converted to five categories (0 days was treated as never; 1–2 days and 3–5 days were treated as rarely; 6–9 days and 10–19 days were treated as sometimes; 20–29 days was treated as most; 30 days was treated as always). Descriptive statistics (percentage) were used to report the percentage of exposures and outcomes, as well as all the control variables; mean (standard deviation) was used to describe BMI and age. The t-test was used to examine sex difference with BMI and age. A Pearson’s chi-squared test was performed to examine the sex difference of all categorical variables and to detect differences in depressive symptoms across various groups (e.g., sex, age group, or AST). Multivariable logistic regression was performed to explore the relationship of AST (reference group = passive) with no depressive symptoms while controlling potential factors (age: reference group = girl; age group: reference group = grade 9; weight status: reference group: obesity; ST: reference group = excessive; smoking: reference group = yes; drinking: reference group = yes; friends: reference group = none). A sex- or age-stratified regression model was also replicated on the basis of the overall model, except that sex or age was excluded as the control variable. Results of the logistic regression were presented as odds ratios (ORs) with 95% confidence intervals (CIs); *p* < 0.05 was set as the significant statistical level. Statistical analyses were performed with SPSS 24.0 (IBM Corp, Chicago, IL, USA).

## 3. Results

Table 1 shows the characteristics of the samples of this study. In total, 6478 students (boys: 46.2%, girls: 53.8%) from Grade 7 to 9 participated in this survey. The numbers of samples decreased with grade increase (*p* < 0.001). The average BMI of participants was 19.1 ± 3.9 with gender difference (boys: 19.4 ± 3.9, girls: 18.8 ± 3.5, *p* < 0.001). According to the BMI, 9.3% were classified as obese, and the proportion of boys was more than that of girls (boys: 12.0%, girls: 7.0%, *p* < 0.001); 79.3% of participants had limited ST without gender difference. In terms of smoking and drinking status, 9.6% and 17.4% of boys reported that they were currently smoking and drinking, respectively, which was greater than the percentage of girls (1.6% for smoking, 10.0% for drinking, both *p* < 0.001). Most students (93.1%) stated that they had friends, and more than half the students (53.2%) stated that they go to school in active ways; there were similar proportions for boys and girls. Similarly, 19.2% of students self-reported that they exhibited depressive symptoms without gender difference. The majority of participants (94.7%) reported no smoking, and there was a significant sex difference of smoking status (*p* < 0.01). A similar situation was found in drinking in the overall sample (*p* < 0.01). Over 60% of participants (regardless of sex) reported having three or more friends, and there was also a sex difference (*p* < 0.01).

Table 2 presents differences in depressive symptoms exhibited by the groups. Among all participants, 19.3% of boys reported depressive symptoms. A similar situation was observed among girls (19.2%, *p* = 0.95). There was very little difference between the 7th, 8th, and 9th graders (7th graders: 18.5%, 8th graders: 18.9%, 9th graders: 21.0%, *p* = 0.13), and between the obesity and non-obesity groups (obesity: 19.6%, non-obesity: 19.2%, *p* = 0.83). The percentage of students exhibiting depressive symptoms in excessive ST groups was more than that in the limited ST group (22.1% > 18.5%, *p* < 0.001). Across different groups of smoking, drinking, friends, and AST, there was a significant difference in depressive symptoms (all *p* < 0.05).

The results of logistic regression on the relationship of AST with depressive symptoms in the total sample are shown in Table 3. In the fully adjusted model, the results of the regression model exhibited that AST was positively associated with no depressive symptoms (adjusted OR = 1.20, 95%CI: 1.06–1.36). 

Table 4 presents the results of logistic regression on the relationship of AST and control variables with no depressive symptoms by sex. Among boys, AST was associated with no depressive symptoms (adjusted OR = 1.34, 95%CI: 1.11–1.60). However, such a significant association was not observed among girls (adjusted OR = 1.10, 95%CI: 0.92–1.30).

Results regarding the relationships of AST with no depressive symptoms by age group are shown in Table 5. Specifically, having AST was only related with no depressive symptoms among Grade 8 and 9 adolescents (adjusted OR for the former = 1.25, 95%CI: 1.01–1.55; adjusted OR for the latter = 1.30, 95%CI: 1.01–1.68). Drinking and having friends were both significantly associated with no depressive symptoms, irrespective of age. Smoking status was also associated with depressive symptoms among Grade 8 and 9 adolescents. 

## 4. Discussion

This is one of only few investigations that have explored the relationship between AST and mental wellbeing indicators in early adolescents. Primary research findings of the current study showed that approximately 50% of early adolescents in Shanghai are active commuters between school and home. This study also found that early adolescents in Shanghai with AST were more likely (adjusted OR = 1.20) to report no depressive symptoms after controlling for other relevant factors. The relationship of AST with no depressive symptoms differed by sex and age among early adolescents. The current study has been the first to examine the relationship of AST with depressive symptoms for early adolescents with regards to sex and age. Two interesting discoveries were the relationship of AST with depressive symptoms, which was only significant among boys and adolescents of higher grades (8 and 9), rather than girls and lower-grade adolescents (Grade 7). 

Previously published studies of samples of Chinese adolescents [17,34] showed that about half of Chinese adolescents had AST. Such a finding is also supported by data from Western research [35,36]. The inconsistency of AST level among our sample and other populations may be due to the different measures. In addition to this, the decline in AST among Chinese adolescents was illustrated by Yang et al. [34], possibly owing to more rapid urbanization developments. Another reason in explaining the lower levels of AST in Shanghai’s early adolescents is more developed public transportation, including the urban metro and bus systems, which may have decreased adolescents’ AST behavior. Nonetheless, because of the health benefits of AST in young people [15,16,17], it is encouraging to promote AST among early adolescents in Shanghai.

There is currently limited evidence regarding AST and mental health indicators among adolescents [17]. The current study found that early adolescents with AST tended to report no depressive symptoms as compared with those who had passive AST. This finding is consistent with a study by Sun et al. [17], in which the authors suggested that children and adolescents with AST were less likely to have depressive symptoms (adjusted OR = 0.91, 95%CI: 0.83–0.98). Although there were different sample characteristics and measurement differences for depressive symptoms between the current study and that of Sun et al. [17], both confirmed a positive role of AST in improving mental health among young people. To explain this interesting and relatively novel finding, some plausible interpretations should be considered. First, AST is a form of PA that can interact with the social and physical environments. Knowledge and observations to date showed that early adolescents who had AST were normally accompanied by their parents, grandparents, friends, or classmates, which raised the safety of travel during the trip between home and school. This, in turn, creates a social environment that has more interactions with other people [16]. A qualitative study by Fusco et al. [37] found that adolescents with AST had greater opportunities to “reflect” on their surrounding environment, which offered more environmental interactions and led to reduced negative emotions. AST happens in outdoor environments that can create a positive mood [16]. Such a positive environment can engender more feelings of happiness, relaxation, or excitement in adolescents, which, in turn, can decrease negative emotions [13], such as depression. Another possible explanation is the stress of adolescents. Being stressed was identified as an important factor for depressive symptoms among adolescents [28]. During this stage, adolescents are more exposed to heavy academic performance and load, so their stress is relatively greater. A study by Lambiase et al. [38] indicated that AST was negatively associated with decreased perceived stress. Consequently, AST may play a role in decreasing depressive symptoms. Conversely, compared with active AST participants, those who had passive AST (e.g., private cars, public bus, or the underground) could be tired or rushed owing to traffic jams. This experience can engender feelings of stress or frustration among adolescents, which might explain why early adolescents with passive AST reported a higher proportion of depressive symptoms [13].

From the perspective of physiology, AST, especially cycling, is linked with improved fitness levels among adolescents [11]. An enhanced fitness level among adolescents can help regulate stress responses, such as the declined secretion of hormones [38]. It has also been documented that enhanced fitness levels can be a protective factor against depressive symptoms among adolescents. Indeed, as an important source of PA, AST is positively associated with higher levels of PA among adolescents [11]. The role of PA in decreasing depressive symptoms was demonstrated [39,40]. Therefore, it is possible that adolescents with AST were more likely to have higher levels of PA, thus reducing depressive symptoms. 

This study found that age and sex differences existed in the relationship of AST with depressive symptoms among early adolescents, which could be viewed as a novel finding. Owing to the extremely limited evidence across the previous literature, there are currently insufficient data for comparison with the findings in this study. Our study’s current data may also not be sufficient to explain sex and age differences in the relationship of AST with depressive symptoms. However, some plausible explanations should be provided. For example, a recent study by Pizarro et al. [36] indicated that boys had more friends during AST as compared with girls. It is possible that more friends can lead to more frequent social interactions, which, in turn, reduces depressive symptoms.

However, the above-mentioned analysis is speculative and cannot be supported by this present study. Hence, to better understand sex and age differences in the relationship of AST with depressive symptoms, more factors should be considered. According to an integrative review [13], the authors suggested that AST can influence mental wellbeing in three ways among adolescents: access, intrinsic (e.g., during travel), and external (e.g., transport by whom). This theoretical framework may be useful to explain sex and age differences in the relationship of AST with depressive symptoms among early adolescents. Future studies should further explain sex and age differences in AST, like trips, attitudes, experiences, or emotions, which, in turn, better explains differences in the relationship of AST with depressive symptoms. On the basis of the above-mentioned analysis, this study offers practical implications that aim at preventing depressive symptoms among early adolescents. First, depressive symptoms could be reduced by effective AST interventions among early adolescents. Second, although AST may have the potential to reduce depressive symptoms among early adolescents, sex- and age-specific mental health promotion actions for reducing depressive symptom are essential.

## 5. Study Limitations and Strengths 

Although this study is one of very few current studies to explore the positive roles of AST on mental health among early adolescents, some limitations must be noted. First, this study adopted a cross-sectional study design, which is inadequate to establish cause-and-effect relationships. Therefore, future studies should use a longitudinal or experiment design to determine the relationship. Second, the variables of depressive symptoms were assessed by a single self-reported question, and its sensitivity and specificity were unclear. In this regard, this self-reported measure can lead to errors on the accurate assessment of the depressive symptoms of participants. Third, it is commonly acknowledged that depressive symptoms among adolescents are associated with more sociodemographic factors, such as parental education and income. Unfortunately, owing to some conditions, this information could not be obtained through measurements, which inevitably influenced the robustness of evidence provided by the current study. Fourth, to adequately assess AST level, a self-reported question was used for measurement. Such a question can only assess the frequency of AST. Indeed, more information regarding AST, like types of AST (walking or cycling), duration, and trips, may also highlight potential factors influencing depressive symptoms. For example, walking or cycling may result in different effects on young people’s physical and mental health indicators. Consequently, it is recommended that future studies use a combination of objective and subjective measures to capture more information on AST. Similarly, our study only selected adolescents of Grades 7–9 as study participants, which may inhibit the understanding of age difference in the relationship of AST with depressive symptoms among adolescents. In addition, the current survey was conducted in Shanghai’s urban areas, so the generalization of the research findings requires caution when applying them to other regions. Shanghai is a large metropolitan area with high urbanization levels and a unique environment (e.g., social and physical) that totally differs from other cities. AST and depressive symptoms among early adolescents are both influenced by the environment [37]. Thus, these research findings are limited to Shanghai’s urban regions.

However, this study still has some strengths. As stated, this study is one of only few to confirm the positive role of preventing depressive symptoms, stressing the importance of AST with mental health among adolescents. The large sample of the study is also a strength. This is helpful for mental health interventions and provisions, at least among adolescents in Shanghai’s urban areas.

## 6. Conclusions

Thus, this study offers evidence that shows that AST can play a role in preventing depressive symptoms among early adolescents in Shanghai’s urban areas. For public health and benefits, mental health issue preventions can incorporate AST as a vital component to increase the effectiveness of interventions, which could give rise to better outcomes among early adolescents. More studies using improved study designs are encouraged to further confirm and elaborate on the relationship of AST with depressive symptoms and explore its underlying mechanisms. 

## Figures and Tables

**Table 1:** Characteristics of Samples in this study.

		Total		Boy		Girl		*p Value*
		n	%	n	%	n	%	
Total		6478	100.0	2990	46.2	3488	53.8	/
Grade group								
	7th	2766	42.7	1235	41.3	1531	43.9	0.00
	8th	2187	33.8	993	33.2	1194	34.2	
	9th	1525	23.5	762	25.5	763	21.9	
Age (mean ± SD)		13.6		13.7		13.6		0.46
BMI (mean ± SD)		19.1±3.7		19.4±3.9		18.8±3.5		0.00
Weight Status								
	Non-Obesity	5875	90.7	2631	88.0	3244	93.0	0.00
	Obesity	603	9.3	359	12.0	244	7.0	
ST								
	Excessive	1342	20.7	607	20.3	735	21.1	0.45
	Limited	5136	79.3	2383	79.7	2753	78.9	
Smoking status								
	Never	6136	94.7	2704	90.4	3432	98.4	0.00
	Rarely	191	2.9	152	5.1	39	1.1	
	Sometimes	81	1.3	72	2.4	9	0.3	
	Most	22	0.3	18	0.6	4	0.1	
	Always	48	0.7	44	1.5	4	0.1	
Drinking								
	Never	5609	86.6	2470	82.6	3139	90.0	0.00
	Rarely	705	10.9	410	13.7	295	8.5	
	Sometimes	113	1.7	72	2.4	41	1.2	
	Most	22	0.3	17	0.6	5	0.1	
	Always	29	0.4	21	0.7	8	0.2	
Friends								
	0	445	6.9	204	6.8	241	6.9	0.00
	1	749	11.6	327	10.9	422	12.1	
	2	1139	17.6	429	14.3	710	20.4	
	3 or more	4145	64.0	2030	67.9	2115	60.6	
AST								
	Passive	3030	46.8	1375	46.0	1655	47.4	0.24
	Active	3448	53.2	1615	54.0	1833	52.6	
Depressive symptom								
	Yes	1246	19.2	576	19.3	670	19.2	0.95
	No	5232	80.8	2414	80.7	2818	80.8	

ST: sedentary time; AST: active school travel; BMI: body mass index; SD: standard deviation.

**Table 2:** Differences in depressive symptom by groups.

		Depressive Symptom				*p Value*
		Yes		No		
		n	%	n	%	
Sex						
	Boy	576	19.3	2414	80.7	0.95
	Girl	670	19.2	2818	80.8	
Grade group						
	7th	512	18.5	2254	81.5	0.13
	8th	414	18.9	1773	81.1	
	9th	320	21.0	1205	79.0	
Weight Status						
	Non-obesity	1128	19.2	4747	80.8	0.83
	Obesity	118	19.6	485	80.4	
ST						
	Excessive	296	22.1	1046	77.9	0.00
	Limited	950	18.5	4186	81.5	
Smoking status						
	Never	1138	91.3	4998	95.5	0.00
	Rarely	52	4.2	139	2.7	
	Sometimes	28	2.2	53	1.0	
	Most	8	0.6	14	0.3	
	Always	20	1.6	28	0.5	
Drinking						
	Never	1005	80.7	4604	88.0	0.00
	Rarely	179	14.4	526	10.1	
	Sometimes	44	3.5	69	1.3	
	Most	8	0.6	14	0.3	
	Always	10	0.8	19	0.4	
Friends						
	0	129	10.4	316	6.0	0.00
	1	157	12.6	592	11.3	
	2	227	18.2	912	17.4	
	3 or more	733	58.8	3412	65.2	
AST						
	Passive	624	20.6	2406	79.4	0.01
	Active	622	18.0	2826	82.0	

ST: sedentary time; AST: active school travel.

**Table 3:** Relationships of AST and control variables with no depressive symptom.

Variables		No Depressive Symptom		
		Adjusted OR	95% CI	
Sex				
	Boy	ref		
	Girl	0.93	0.82	1.06
Age group				
	7th	ref		
	8th	1.00	0.87	1.16
	9th	0.92	0.78	1.08
Weight Status				
	Non-obesity	ref		
	Obesity	0.97	0.78	1.20
ST				
	Excessive	ref		
	Limited	1.15	0.99	1.34
Smoking status				
	never	ref		
	rarely	0.74	0.53	1.05
	sometimes	0.54	0.33	0.87
	most	0.55	0.23	1.35
	always	0.46	0.25	0.86
Drinking				
	never	ref		
	rarely	0.69	0.57	0.84
	sometimes	0.43	0.29	0.65
	most	0.50	0.20	1.23
	always	0.52	0.23	1.16
Friends				
	0	ref		
	1	1.54	1.17	2.02
	2	1.61	1.25	2.07
	3 or more	1.89	1.51	2.36
AST				
	Passive	ref		
	Active	1.20	1.06	1.36

ST: sedentary time; excessive: more than 2 hours of ST per day; limited: no more than 2 hours of ST per day; ref: reference group; OR: odds ratio; CI: confidence interval.

**Table 4:** Relationships of AST and control variables with no depressive symptom by sex.

Variables		No Depressive Symptom					
		Boy			Girl		
		Adjusted OR	95%CI		Adjusted OR	95%CI	
Grade group							
	7th	ref			ref		
	8th	1.15	0.92	1.43	0.92	0.75	1.11
	9th	0.95	0.76	1.20	0.90	0.72	1.13
Weight Status							
	Non-obesity	ref			ref		
	Obesity	1.00	0.75	1.32	0.95	0.68	1.32
ST							
	Excessive	ref			ref		
	Limited	0.96	0.77	1.21	1.31	1.07	1.60
Smoking status							
	never	ref			ref		
	rarely	0.78	0.52	1.17	0.54	0.27	1.06
	sometimes	0.53	0.31	0.89	0.28	0.07	1.10
	most	0.55	0.20	1.53	0.33	0.05	2.36
	always	0.46	0.24	0.88	0.24	0.02	2.68
Drinking							
	Never	ref			ref		
	Rarely	0.74	0.57	0.96	0.66	0.50	0.88
	Sometimes	0.72	0.41	1.27	0.20	0.10	0.38
	Most	0.53	0.19	1.49	0.55	0.08	3.64
	Always	0.39	0.16	0.97	1.30	0.16	10.59
Friends							
	0	ref			ref		
	1	1.50	1.00	2.26	1.53	1.06	2.21
	2	1.75	1.18	2.59	1.51	1.08	2.11
	3 or more	1.78	1.28	2.48	1.94	1.43	2.63
AST							
	Inactive	ref			ref		
	Active	1.34	1.11	1.61	1.10	0.92	1.30

ST: sedentary time; excessive: more than 2 hours of ST per day; limited: no more than 2 hours of ST per day; ref: reference group; OR: odds ratio; CI: confidence interval; bold fonts denote statistical significance.

**Table 5:** Relationships of AST and control variables with no depressive symptoms by age group.

Variables		No Depressive Symptoms								
		7th			8th			9th		
		Adjusted OR	95%CI		Adjusted OR	95%CI		Adjusted OR	95%CI	
Sex										
	Boy	ref			ref			ref		
	Girl	1.03	0.84	1.25	0.79	0.63	1.00	0.97	0.74	1.26
Weight Status										
	Non-obesity	ref			ref			ref		
	Obesity	0.71	0.52	0.99	1.17	0.79	1.72	1.31	0.84	2.05
ST										
	Excessive	ref			ref			ref		
	Limited	1.20	0.95	1.53	1.25	0.97	1.61	0.96	0.71	1.30
Smoking status										
	never	ref			ref			ref		
	rarely	1.07	0.54	2.12	0.82	0.48	1.42	0.47	0.26	0.85
	sometimes	0.38	0.16	0.91	0.80	0.28	2.26	0.55	0.26	1.15
	most	0.21	0.03	1.35	1.03	0.11	9.69	0.67	0.19	2.33
	always	0.36	0.07	1.97	0.15	0.05	0.50	0.81	0.33	1.99
Drinking										
	never	ref			ref			ref		
	rarely	0.65	0.47	0.91	0.68	0.50	0.93	0.78	0.54	1.13
	sometimes	0.41	0.20	0.84	0.52	0.27	1.04	0.34	0.16	0.72
	most	0.53	0.06	4.62	0.35	0.10	1.26	0.75	0.15	3.86
	always	1.01	0.19	5.28	0.54	0.14	2.07	0.28	0.06	1.22
Friends										
	0	ref			ref			ref		
	1	1.56	1.01	2.39	1.47	0.90	2.40	1.58	0.93	2.68
	2	1.69	1.13	2.52	1.50	0.95	2.35	1.71	1.04	2.80
	3 or more	1.79	1.27	2.51	1.77	1.18	2.65	2.28	1.47	3.54
AST										
	Passive	ref			ref			ref		
	Active	1.13	0.93	1.37	1.25	1.01	1.55	1.30	1.01	1.68

ST: sedentary time; excessive: more than 2 h of ST per day; limited: no more than 2 hours of ST per day; ref: reference group; OR: odds ratio; CI: confidence interval; bold fonts denote statistical significance.
